# Salvage surgery following downstaging of advanced non‐small cell lung cancer by targeted therapy

**DOI:** 10.1111/1759-7714.14044

**Published:** 2021-06-15

**Authors:** Kuo Li, Xiaonian Cao, Bo Ai, Han Xiao, Quanfu Huang, Zheng Zhang, Qian Chu, Li Zhang, Xiaofang Dai, Yongde Liao

**Affiliations:** ^1^ Department of Thoracic Surgery, Union Hospital, Tongji Medical College Huazhong University of Science and Technology Wuhan China; ^2^ Department of Thoracic Surgery, Tongji Hospital, Tongji Medical College Huazhong University of Science and Technology Wuhan China; ^3^ Department of Thoracic Surgery The Affliated Yantai Yuhuangding Hospital of Qingdao University Yantai China; ^4^ Department of Oncology, Tongji Hospital, Tongji Medical College Huazhong University of Science and Technology Wuhan China; ^5^ Department of Oncology, Union Hospital, Tongji Medical College Huazhong University of Science and Technology Wuhan China

**Keywords:** advanced NSCLC, salvage surgery, targeted therapy

## Abstract

**Background:**

Advanced non‐small cell lung cancer (NSCLC) accounts for a high proportion of lung cancer cases. Targeted therapy improve the survival in these patients, but acquired drug resistance will inevitably occur. If tumor downstaging is achieved after targeted therapy, could surgical resection before drug resistance improve clinical benefits for patients with advanced NSCLC? Here, we conducted a clinical trial showing that for patients with advanced driver gene mutant NSCLC who did not progress after targeted therapy, salvage surgery (SS) could improve progression‐free survival (PFS). Herein, we retrospectively reviewed our former clinical trial and thoracic cancer database in our medical institutions.

**Methods:**

We identified patients with advanced driver gene mutant NSCLC treated with targeted therapy plus SS or targeted therapy alone in our former clinical trial and our thoracic cancer database from July 2016 to July 2019. PFS was compared between the targeted therapy plus SS group and the targeted therapy only group using the log‐rank test.

**Results:**

We identified 73 patients with driver gene mutant NSCLC who were treated with targeted therapy and 18 treated with targeted therapy plus SS.Among the 18 patients treated with targeted therapy plus SS, there were no obvious perioperative complications and deaths. Targeted therapy followed by SS resulted in a significantly longer PFS compared with targeted therapy alone (23.4 months VS 12.9 months, *p* = 0.0004).

**Conclusions:**

Salvage surgery after tumor downstaging is a promising therapeutic strategy for some patients with advanced (stage IIIB–IV) NSCLC and may offer a new therapeutic option for multidisciplinary comprehensive treatment of lung cancer.

## Graphical abstract

Salvage surgery after tumor downstaging by targeted therapy is a promising therapeutic strategy for some patients with advanced NSCLC.

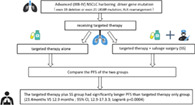

## INTRODUCTION

Lung cancer is a malignant tumor with the highest incidence and mortality worldwide, and non‐small cell lung cancer (NSCLC) accounts for approximately 80% of the total cases of lung cancer. Over 60% of lung cancer patients present with locally advanced or metastatic disease (stage III–IV) at the time of diagnosis, at which point surgical resection may not be an option.^1^, ^2^ For these patients, platinum chemotherapy is the traditional standard treatment instead of surgery. However, it remains poorly controlled with progression‐free survival (PFS) and has severe side effects.^3^ In recent years, molecular targeted therapy have become the first‐line treatment for advanced NSCLC patients harboring a driver gene mutation due to the advantages of better efficacy and minor adverse reactions.^4^

Epidermal growth factor receptor (EGFR) activating mutations have been reported to occur more frequently in patients with NSCLC, especially in Asian populations.^5^ EGFR tyrosine kinase inhibitors (TKIs) have been approved for the treatment of NSCLC patients with sensitive *EGFR* mutations, and patients have been found to have a higher response rate and longer PFS than those administered platinum‐based chemotherapy. However, most patients inevitably develop acquired drug resistance after 8–12 months. Although third generation TKIs such as osimertinib have been developed, acquired resistance is still an existing problem.^6^ Anaplastic lymphoma kinase (ALK), another significant driver gene mutation site, also has a remarkable response when ALK inhibitors are used in patients with advanced ALK‐mutated NSCLC. Patients who are administered ALK inhibitors, such as crizotinib, will also face drug acquired resistance, with a PFS about 10.9 months.^7^

Clearly, more effective strategies to prevent the emergence of resistance are urgently required. It has been reported that some advanced NSCLC patients achieve long‐term survival outcomes when treated with local consolidative therapy (LCT).^8^, ^9^ In addition, some studies have demonstrated that combining LCT with targeted treatment is a valuable therapeutic strategy.^10^, ^11^, ^12^ Most LCTs are radiotherapy rather than surgery, and are mainly directed at metastases. For advanced NSCLC patients harboring sensitive gene mutations, whether the addition of salvage surgery (SS) could improve PFS remains to be explored. To determine the feasibility and potentially effectiveness of SS for advanced NSCLC treated with targeted therapy, we conducted a multi‐institutional, single‐arm clinical trial (chictr‐opc‐17012613).

Analysis of our study results showed an inspiring outcome that this treatment strategy could bring clinical benefits. Herein, we retrospectively reviewed our former clinical trial and thoracic cancer database in our subordinate hospitals (Union Hospital,Tongji Hospital), to determine the clinical outcomes of SS after targeted therapy in patients with advanced driver gene mutant NSCLC.

## METHODS

### Study design

This was a post‐hoc analysis of our registered clinical trial and a retrospective review of the thoracic cancer database, a prospectively collected database that includes patient demographics, tumor profiles, treatment history, and clinical outcomes. PFS, perioperative complications and safety were compared between targeted therapy plus SS and targeted therapy only treated patients. The study was approved by the ethics committee of Tongji Medical College, Huazhong University of Science and Technology (IRB ID 20140609). Informed consent was received from all patients before treatment.

### Patient cohort

We conducted a retrospective review of patients with advanced driver gene mutant NSCLC who were treated with targeted therapy between July 2016 and July 2019. Recruited patients met the following criteria: (i) Pathological diagnosis of NSCLC with confirmed activation of driver gene mutation (*EGFR* mutant: exon 19 deletion or exon 21 L858R mutation; ALK‐rearrangement) by amplification refractory mutation system (ARMS), (ii) stage IIIB–IV according to the eighth edition of the American Joint Committee on Cancer staging system confirmed by pathological diagnosis and positron emission tomography‐computed tomography (PET‐CT) and biopsy, (iii) 18 years or older, (iv) receiving molecular targeted agents as first‐line therapy. Patients treated with targeted therapy without progression and radiological confirmation of tumor downstaging (≤stage IIIA) by PET‐CT followed by SS were enrolled into the group of targeted therapy plus SS. Patients treated with targeted therapy without any other intervention were enrolled into the group of targeted therapy only. The molecular targeted agents used in our study included gefitinib (250 mg, once a day) and crizotinib (250 mg, twice a day). Salvage surgery was defined as surgical intervention based on standard operation (lobectomy plus lymphadenectomy) of NSCLC for advanced patients who initially had no surgical indications, but achieved significant downstaging (≤stage IIIA) without progression after targeted therapy. Targeted therapy was continued after SS until progression. Baseline characteristics, including age at diagnosis (taken at date of diagnostic biopsy), sex, smoking status, mutation type, targeted therapy duration, clinical stage, and postoperative pathological response, were collected from electronic records. Follow‐up information was recorded periodically. PFS was compared between the targeted therapy plus SS group and targeted therapy only group, which was defined from the time of taking oral medicine to progression or death. For patients who remained alive without progression, the PFS date was censored at their last clinic follow‐up.

### Statistical analysis

Data are described using the median and range for continuous variables and as percentages with 95% confidence intervals for qualitative variables. Survival time is described using the Kaplan–Meier method. Statistical analyses were performed using the SPSS 20.0 (IBM SPSS Inc.) and MedCalc (19.0.4 version) software packages.

## RESULTS

### Patient characteristics

In our database, we identified 73 patients with advanced driver gene (exon 19 deletion, L858R, ALK‐rearrangement) mutant NSCLC treated with targeted therapy (targeted therapy only group) and 18 that were treated with targeted therapy followed by SS (targeted therapy plus SS group). The patient characteristics of both groups are shown in Table 1, and the characteristics of the targeted therapy plus the SS group are presented in Table 2.

**TABLE 1:** Patient characteristics of two groups

Characteristic	Targeted therapy + SS	Targeted therapy
Total patients	*N* = 18	*N* = 73
Gender		
Male	9	27
Female	9	46
Age, years		
Median	54	58
Range	32–67	35–77
Smoking history		
Never	13	55
Active/former	5	18
Mutation type		
Exon 19 deletion	11	49
L858R	5	23
ALK	2	1
CS		
IIIB–IIIC	8	21
IV	10	52
Treatment protocols	Performing salvage surgery after targeted therapy	Receiving targeted therapy only

Abbreviations: ALK, anaplastic lymphoma kinase; CS, clinical stage.

**TABLE 2:** Patient characteristics and detailed information in the targeted therapy plus SS group

Case	Gender	Age (years)	Histology	Gene mutation type	CS before targeted therapy	Distant metastasis	Targeted drug	Drug duration	CS after targeted therapy
1	F	63	AD	19‐Del	cT2N2M1c,IV	Liver, Ribs	Gefitinib	9 months	cT2N0M0,IB
2	F	36	AD	19‐Del	cT4N2M1a,IV	Contralateral lung, Pleura	Gefitinib	7 months	cT2N0M0,IB
3	F	59	AD	19‐Del	cT1cN2M1a,IV	Malignant effusion	Gefitinib	7 months	cT1bN0M0,IA
4	M	48	AD	19‐Del	cT2aNxM1a,IV	Multiple lungs	Gefitinib	4 months	cT1N0M0,IA
5	M	58	AD	19‐Del	cT1cNxM1a,IV	Multiple lungs	Gefitinib	3 months	cT1bN0M0,IA
6	M	51	AD	19‐Del	cT4N2M0,IIIB	—	Gefitinib	2 months	cT1N0M0,IA
7	M	32	AD	19‐Del	cT1cN3M0,IIIB	—	Gefitinib	2 months	cT1bN0M0,IA
8	M	45	SC	ALK	cT3N3M0,IIIC	—	Crizotinib	2 months	cT1N0M0,IA
9	F	59	ADCA	19‐Del	cT2N3M0,IIIB	—	Gefitinib	2 months	cT1N0M0,IA
10	M	48	AD	L858R	cT2N2M1b,IV	Ilium	Gefitinib	2 months	cT1N0M0,IA
11	F	59	AD	L858R	cT4N0M1a,IV	Multiple lung	Gefitinib	2 months	cT1N0M0,IA
12	F	67	AD	ALK	cT3N2M0,IIIB	—	Crizotinib	2 months	cT1N0M0,IA
13	F	55	AD	19‐Del	cT2N2M1a,IV	Contralateral lung	Gefitinib	2 months	cT1N0M0,IA
14	M	54	AD	L858R	cT3N2M0,IIIB	—	Gefitinib	2 months	cT1N0M0,IA
15	M	56	AD	19‐Del	cT3N2M0,IIIB	—	Gefitinib	2 months	cT1N0M0,IA
16	M	60	AD	19‐Del	cT3N3Mx,IIIC	—	Gefitinib	2.5 months	cT2N0M0,IB
17	F	65	AD	L858R	cT2N2M1a,IV	Contralateral lung	Gefitinib	12 months	cT2N0M0,IB
18	F	61	AD	L858R	cT2N2M1a,IV	Contralateral lung	Gefitinib	3 months	cT1N0M0,IA

Abbreviations: AD, adenocarcinoma; ADCA, adenosquamous carcinoma; CS, clinical stage; F, female; M, male; SC, squamous carcinoma.

**FIGURE 1:**
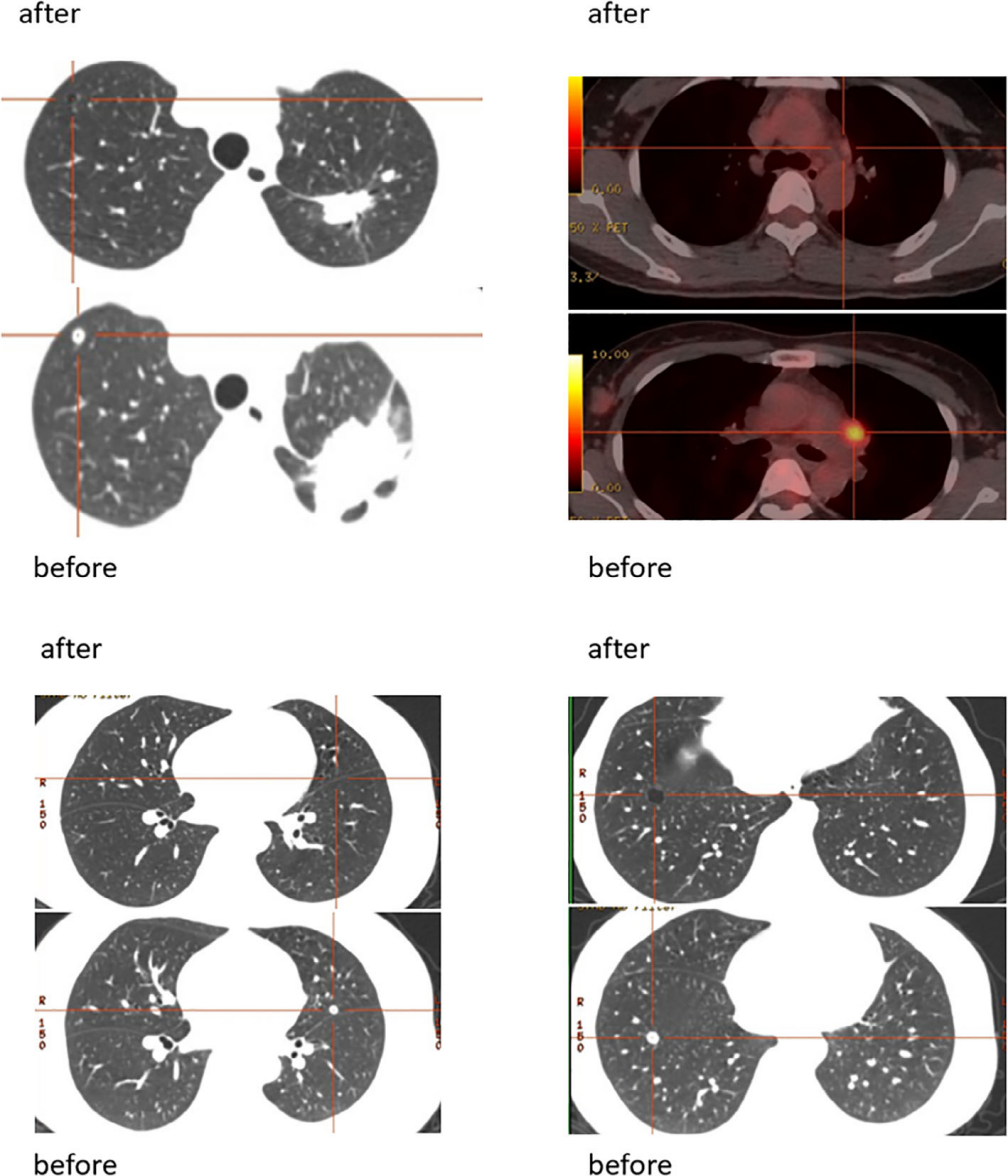
Positron emission tomography‐computed tomography (PET‐ CT) before and after targeted therapy in Case 2

**FIGURE 2:**
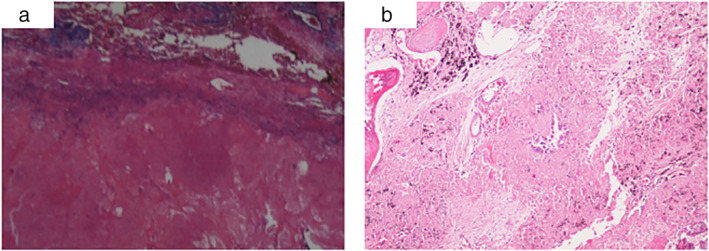
(a) The primary pulmonary lesion achieved a complete pathological response. (b) A major pathological response (MPR) was defined as the identification of 10% or less of residual viable tumor cells in the resected primary tumor

Of the 18 patients, there were 16 cases of adenocarcinoma (88.9%), and the median age was 54 years. There were nine males (55.0%), 10 cases of stage IV (55.6%), nine (50%) had a history of nonsmoking, and 16 cases (88.9%) had sensitive *EGFR* mutation. Radiographic evaluation showed significant tumor downstaging (Case 2, Figure 1). Patients underwent lobectomy and lymphadenectomy (Table 3). The lymph nodes had become a thickened fibrous scar in some patients, and it was difficult to expose vessels. There were eight cases of postoperative stage IA, one case of postoperative stage IB, and four cases of postoperative stage IIIA. Pathologically, the postoperative results showed that two patients reached pathological complete response (pCR), and MPR (major pathologic response: defined as the identification of 10% or less of residual viable tumor cells in the resected primary tumor) occurred in eight patients (44.4%) (Figure 2).

**TABLE 3:** Patient details of surgery and postoperative information in the targeted therapy plus SS group

Case	Mode of surgery	Postoperative CS	Adjuvant therapy	Final outcome
1	VATS lobectomy + SML	pT2N0M0R0,IB	Gefitinib (13 months)	Progression of Pubic bone metastasis
2	VATS lobectomy + SML	pT2N2M0R0,IIIA	Gefitinib (6 months)	Progression of intrapulmonary metastasis
3	VATS lobectomy + SML	pT1bN0M0R0,IA	Gefitinib (13 months)	Progression of Pubic bone metastasis
4	VATS lobectomy + SML	pT1N2M0R0,IIIA	Gefitinib (12 months)	Progression‐free
5	VATS lobectomy + SML	pT1N0M0R0,IA	Gefitinib (17 months)	Progression‐free
6	VATS lobectomy + SML	pT1N0M0R0,IA	Gefitinib (17 months)	Progression of intrapulmonary metastasis
7	VATS lobectomy + SML	pT1bN0M0R0,IA	Gefitinib (10 months)	Progression of supraclavicular lymph nodes
8	VATS lobectomy + SML	pCR	Crizotinib (12 months)	Progression of intracranial metastasis
9	VATS lobectomy + SML	pT1cN0M0R0,IA	Gefitinib (29 months)	Progression of brain metastasis
10	VATS lobectomy + SML	pT1bN0M0R0,IA	Gefitinib (27 months)	Progression‐free
11	VATS lobectomy + SML	pT1bN0M0R0,IA	Gefitinib (25 months)	Progression‐free
12	VATS lobectomy + SML	pT1aN0M0R0,IA	Crizotinib (24 months)	Progression‐free
13	VATS lobectomy + SML	pT1bN0M0R0,IA(MPR)	Gefitinib (24 months)	Progression‐free
14	VATS lobectomy + SML	pT1aN2M0R0,IIIA	Gefitinib (14 months)	Progression‐free
15	VATS lobectomy + SML	pT1bN0M0R0,IA	Gefitinib (28 months)	Progression‐free
16	VATS lobectomy + SML	pCR	Gefitinib (8.5 months)	Progression of bone metastasis
17	Sleeve lobectomy + SML	pT2N2M0R0,IIIA	Gefitinib (4 months)	Progression of intrapulmonary metastasis
18	VATS lobectomy + SML	pT1aN0M0R0,IA(MPR)	Gefitinib (3 months)	Progression‐free

Abbreviations: MPR, major pathological response; pCR, pathological complete response; SML, systematic mediastinal lymphadenectomy; VATS, video‐assisted thoracoscopic surgery.

### Perioperative complications

No patient had grade 4 adverse events or died because of an adverse event. Video‐assisted thoracoscopic surgery (VATS) was successfully performed and only one patient was converted to thoracotomy because a sleeve lobectomy was required. One patient in the targeted therapy plus SS group had postoperative chylothorax. There were no other obvious perioperative complications, such as pulmonary leakage, pulmonary infection, cardiac insufficiency, and liver or kidney dysfunction. Compared with conventional surgery of lung cancer in our group, salvage surgery did not increase blood loss, drainage time, and hospital stays. The drainage time in the group of targeted therapy plus SS was (2.7 ± 1.2) days, and the hospital stay was (9.9 ± 1.5) days. All patients had good wound healing, and no perioperative deaths occurred.

### Follow‐up

**FIGURE 3:**
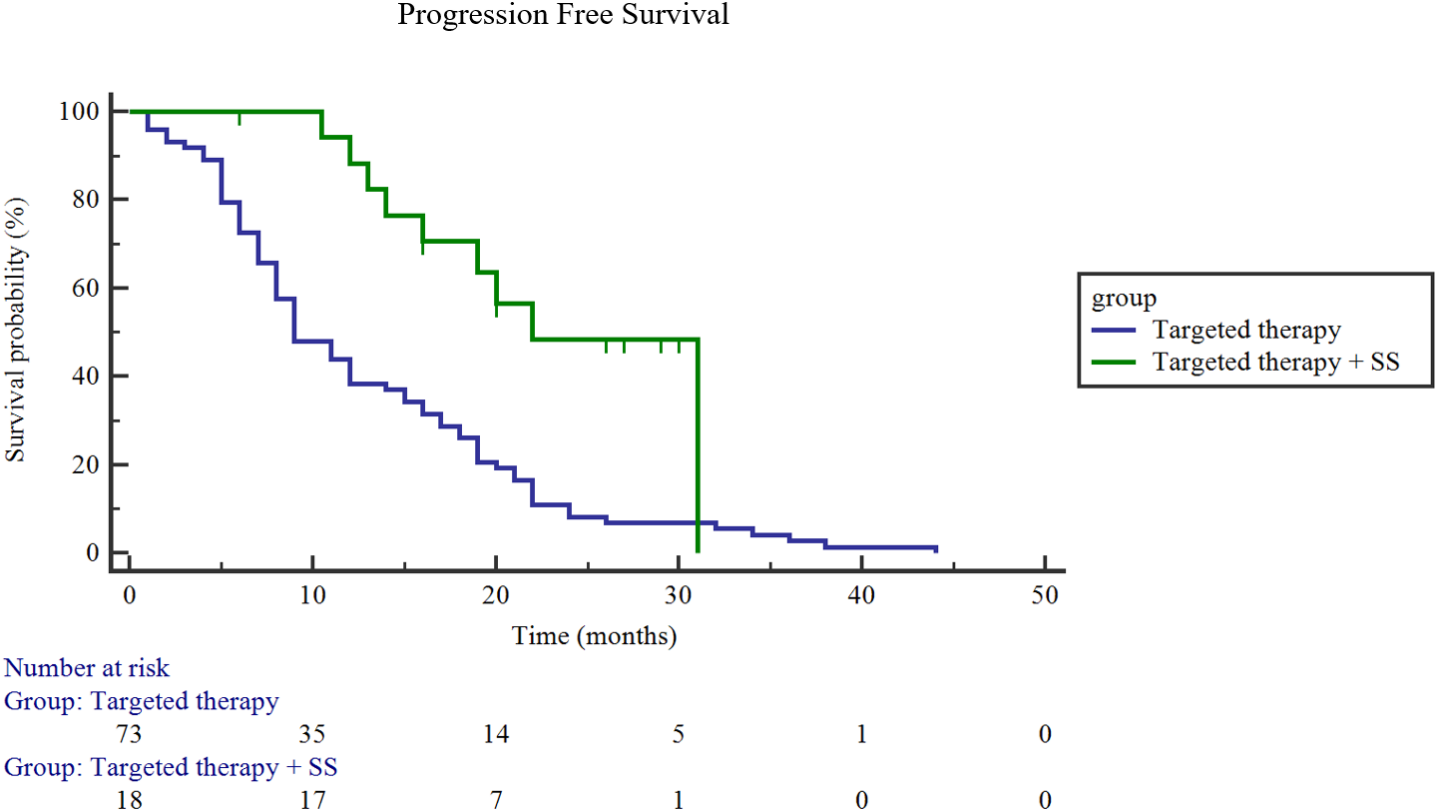
Progression‐free survival (PFS) between the targeted therapy plus SS group and the targeted therapy only group. The median PFS in the targeted therapy plus SS group: 23.4 months; targeted therapy only group: 12.9 months; (95% CI, 12.9–17.3.3; Log rank, *p* = 0.0004)

**FIGURE 4:**
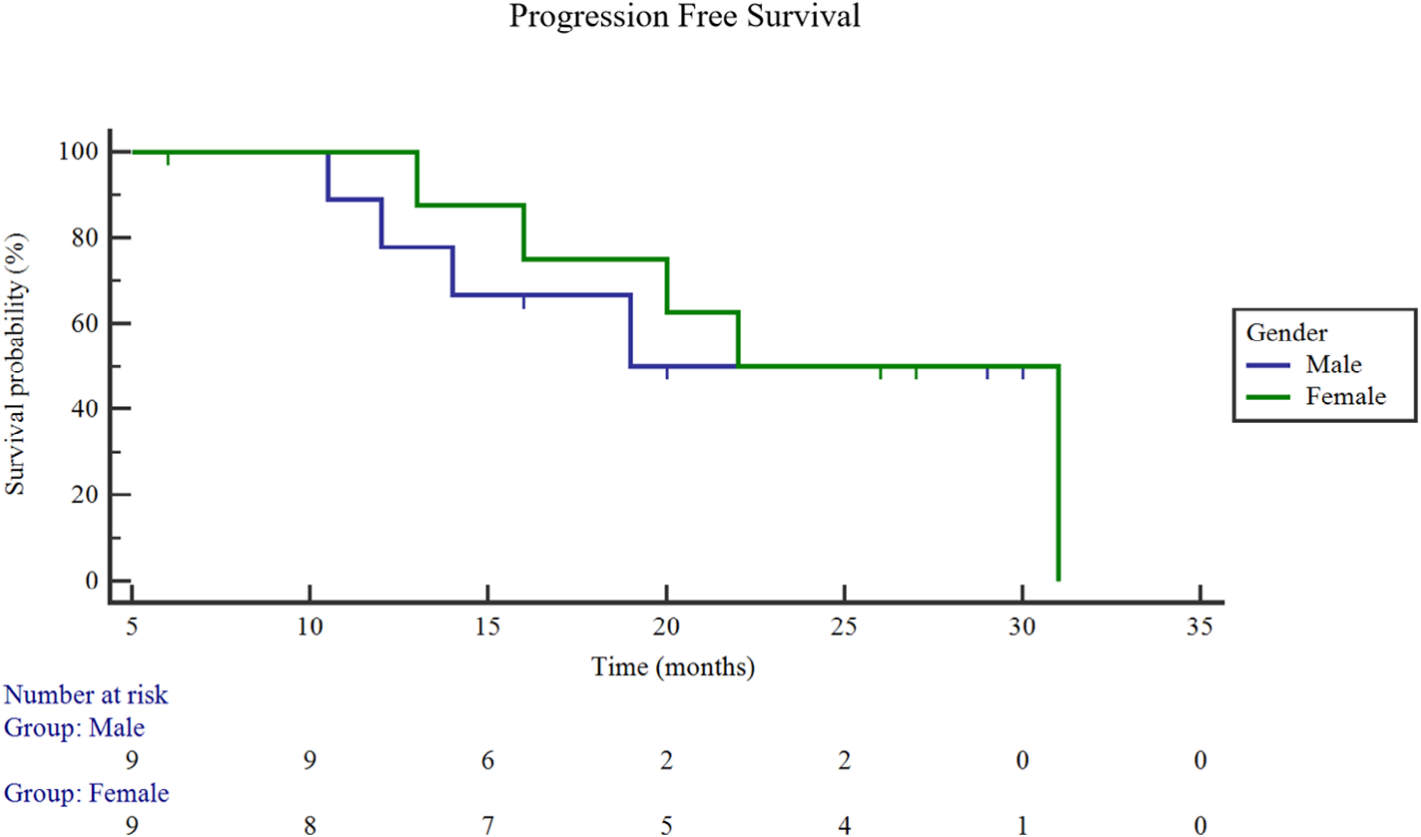
Progression‐free survival (PFS) between male and female patients in the targeted therapy plus SS group. PFS of male patients: 22.2 months; the PFS of female patients: 24.4 months; *p* = 0.71

**FIGURE 5:**
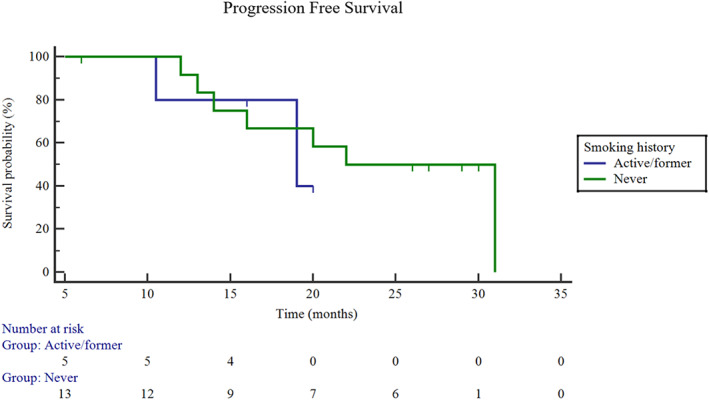
Progression‐free survival (PFS) between smokers and never‐smokers in the targeted therapy plus SS group. PFS of smokers: 17.7 months; the PFS of nonsmokers: 23.6 months; *p* = 0.80

**FIGURE 6:**
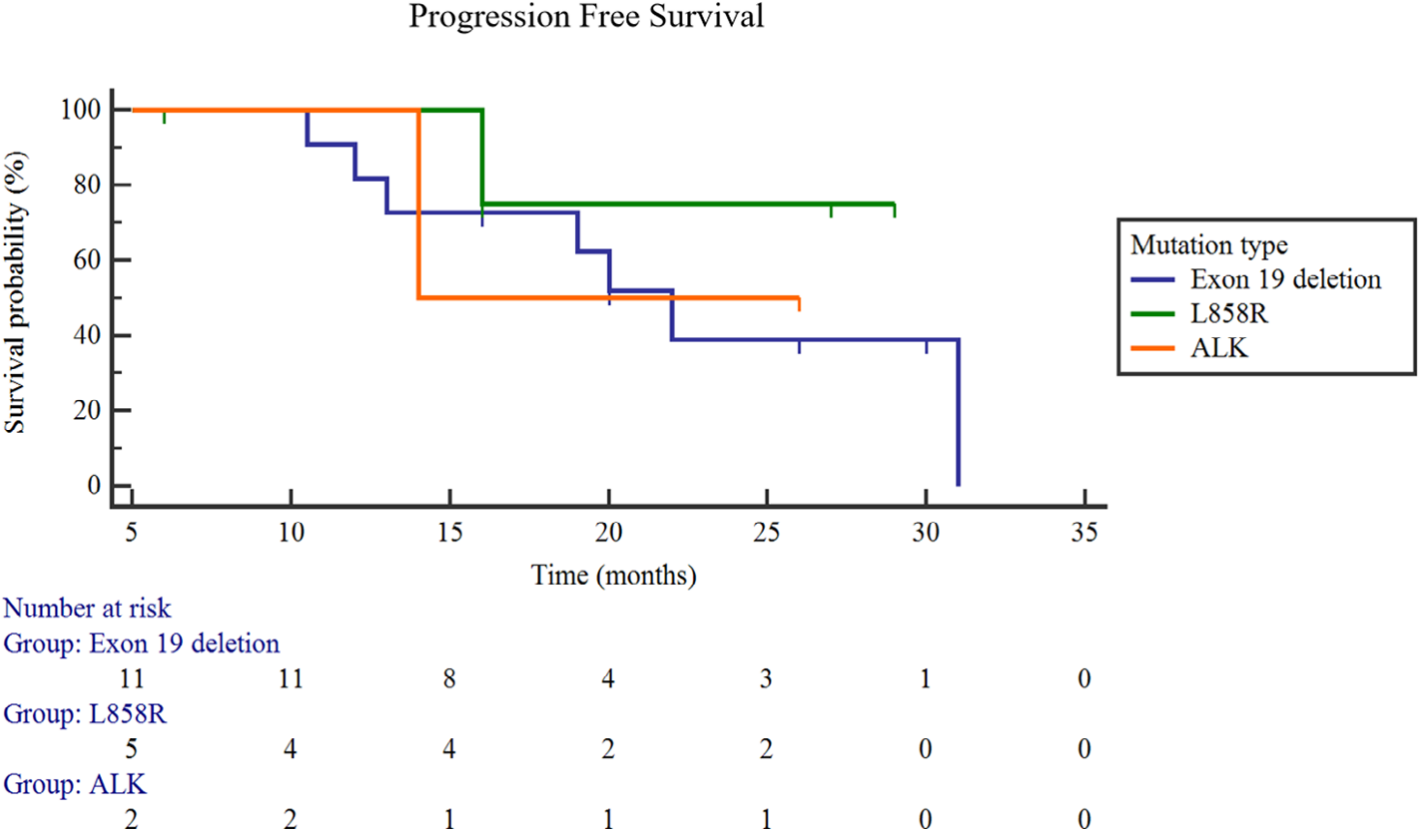
Progression‐free survival (PFS) among different mutation types. Exon 19 deletion: 22.2 months; L858R: 25.6 months; ALK‐rearrangement: 20.0 months; *p* = 0.69

In December 2019, all patients received their last follow‐up. Patients treated with targeted therapy plus SS had significantly longer PFS than patients treated with targeted therapy only. The median PFS was 23.4 months in the targeted therapy plus SS group and 12.9 months in the targeted therapy only group (95% CI: 12.9–17.3.3; Log rank *p* = 0.0004) (Figure 3). With a median follow‐up in the targeted therapy plus SS group of 27.5 months (ranging from 6.0 to 41.0 months), the overall survival (OS) was not reached. Nine patients in the targeted therapy plus SS group had disease progression because of new metastasis, but there was no local recurrence. We further performed subgroup analysis for the SS group. The PFS of male patients was 22.2 months and that of females was 24.4 months (Figure 4), but the difference was not statistically significant (*p* = 0.71). The PFS of smokers was 17.7 months, and the PFS of never‐smokers was 23.6 months (Figure 5), while there was also no statistically significant difference between them (*p* = 0.80). The PFS was also different among different mutation types (exon 19 deletion: 22.2 months; L858R: 25.6 months; ALK‐rearrangement: 20.0 months; *p* = 0.69) (Figure 6).

## DISCUSSION

Given the obvious advantages of targeted therapy in patients with advanced NSCLC, we deliberated whether we could perform salvage surgery following downstaging to provide a treatment model worth exploring. It has previously been reported that salvage surgery was used in unresectable hepatocellular carcinoma (HCC),^13^, ^14^ and some advanced HCC patients achieved long‐term survival from this model, while salvage surgery has rarely been reported in patients with advanced NSCLC. Although there have been sporadic case reports about NSCLC indicating that this model could also improve survival benefit,^15^, ^16^, ^17^, ^18^, ^19^ the current evidence is insufficient to illustrate its feasibility and effectiveness. In addition, some reports have reached the opposite conclusion. A retrospective study in Japan with nine cases found that this model had no obvious survival advantage because PFS was only six months, and patients had poor wound healing.^19^

Compared with the Japanese study by Hishida et al., our study had a relatively large number of cases (18 cases), and a longer PFS. We observed that salvage surgery following downstaging of advanced NSCLC was feasible and valuable. Our encouraging results were consistent with previous case reports but different from the Japanese study.^19^ These discrepancies may be due to the following: (i) All our patients were examined by PET‐CT prior to surgery to assess their general condition, which meant that our staging assessment was more accurate. However, there was no mention of PET‐CT examination in the Japanese study, and postoperative pathology showed that most patients (7/9, 77.8%) were later stage, which further indicated the deviation of preoperative staging. (ii) Our preoperative medication duration was four months (2–12 months) on average, and we intervened before drug resistance developed. In the Japanese study, the mean duration was 17 months (2–36 months), and three cases had already progressed to drug resistance during surgery. Prolonged duration may increase the risk of drug resistance and further affect the PFS.

So why could this model benefit patients? We deemed that although the preoperative targeted therapy in this model could not be called neoadjuvant therapy, its concept and effect was similar: (i) Shrink the primary tumor to downstage it, (ii) improve surgical resectability, (iii) eliminate or inhibit possible preoperative micrometastasis, and (iv) maximize the risk reduction for postoperative progression.

Previous studies have confirmed that patients with stage III NSCLC can benefit from neoadjuvant targeted therapy.^20^, ^21^ These studies reflect the value of combining targeted therapy with surgery. There have been reports indicating that some advanced NSCLC, such as oligometastatic status, is an intermediate state between a local primary tumor and extensive metastasis, and LCT can benefit patients.^10^, ^11^, ^12^, ^22^, ^23^, ^24^, ^25^ Among these reports, it has been demonstrated that targeted drugs plus LCT may be a better therapeutic option compared with targeted therapy alone, with patients surviving for a longer period.^22^, ^23^, ^24^ In addition, an ongoing clinical trial (NCT03410043) used three‐generation targeted drugs combined with LCT in the treatment of advanced NSCLC,^22^ suggesting that the treatment model may be a hot topic of great interest, and have potential clinical application value. Our study extended this therapeutic strategy and focused on exploring the value of salvage surgery, which is different from other studies.

Several studies have highlighted that during the failure of targeted therapy in patients with advanced NSCLC, recurrence of the primary tumor in the lung is the most significant risk factor and reducing primary tumor lesions may be an important step in prolonging PFS.^26^, ^27^ Local treatment for primary lesions includes radiotherapy, interventional ablation, and surgery. At present, radiotherapy is the primary treatment. although its failure is still reported in patients treated locally with radiotherapy. We conclude that surgery may be superior to radiotherapy as LCT. The reasons for this are as follows: (i) Although targeted therapy can achieve downstaging, a complete pathological response is only achieved in a few patients, and most patients still have residual tumor lesions. Compared with radiotherapy, surgery can completely remove the primary tumor, the most important factor affecting efficacy, which can minimize the residual tumor and reduce recurrence risk. (ii) High EGFR expression in many tumors may lead to radiotherapy insensitivity and reduce tumor control rate, resulting in tumor recurrence.^28^ (iii) Radiotherapy will increase the heterogeneity in the residual tumor tissue. The drug‐resistant cloning caused by radiotherapy will occur in the residual lesions, which promotes locoregional recurrence. Surgical therapy may be a better choice because it can reduce the risk of recurrence and acquired resistance by completely removing primary tumor lesions.^29^ (iv) Combining targeted therapy with radiotherapy will increase the incidence of radiation pneumonia to further decrease survival rates. (v) We can obtain the tissue specimens from surgery following targeted therapy to further evaluate treatment efficacy through postoperative pathology and exploit specimens to study their molecular mechanisms.

In our study, we found that PFS was longer in female and never‐smoker patients, which was also consistent with the dominant group of targeted therapy, but the differences were not statistically significant, whereas patients with L858R had better PFS among different mutation types, which is different from the literature. This may be related to our small sample size. As an exploratory treatment, the preoperative drug duration currently have no unified provision. According to our experience, after 4–6 months of oral administration may be the best time to perform SS before drug resistance occurs, because the tumor burden is controlled better at this time. Of course, more cases need to be accumulated to further explore and analyze this treatment in order to choose which patient can benefit best from SS.

Although these findings are valuable, there are several limitations that should be acknowledged. First, this was a retrospective study, and our clinical trial was a nonrandomized, single arm study, with potential selection bias. In addition, the number of patients enrolled in the targeted therapy plus SS group was relatively small. Furthermore, there are still many questions that need to be further examined, such as the accuracy of staging, variability in timing of targeted therapy receipt, variability in postoperative continuation of targeted therapy, and the heterogeneity of this group and so on. During surgery, for example, we found that a small proportion of patients had obvious fibrosis which may have been related to targeted therapy and increased the difficulty of lymphadenectomy. Although we performed systematic lymphadenectomy if intraoperative conditions permitted instead of taking samples so as to minimize the tumor burden, the surgical options require further discussion. On the other hand, identification of patient subgroups most likely to derive a therapeutic benefit from salvage surgery remains challenging. We believe that surgery is still necessary even if pCR is achieved. In future, we plan to address these issues via second biopsy or liquid biopsy detection such as ctDNA. For further exploration, we will register a prospective randomized study in order to obtain stronger evidence.

In conclusion, this type of strategy is valuable. Our analysis shows that patients with driver gene mutant NSCLC tolerated SS without obvious adverse events and had a promising PFS compared with the control group treated with targeted therapy alone, which could provide a new option for multidisciplinary comprehensive treatment of lung cancer.

## CONFLICT OF INTEREST

None declared.
